# XVIII Reunión Post-ECTRIMS: Revisión de las Novedades Presentadas en el Congreso ECTRIMS 2025 (Parte I)

**DOI:** 10.31083/RN51701

**Published:** 2026-08-14

**Authors:** Óscar Fernández, Xavier Montalban, Adrián Arés, Eduardo Agüera, Yolanda Aladro, Ana Alonso, Rafael Arroyo, Luis Brieva, Carmen Calles, Ana Belén Caminero, Tamara Castillo-Triviño, Lucienne Costa-Frossard, Sara Eichau, Lamberto Landete, Miguel Llaneza, Sara Llufriu, José E. Meca-Lallana, Virginia Meca-Lallana, Enric Monreal, Ester Moral, Celia Oreja-Guevara, José María Prieto, Lucía Romero-Pinel, Àlex Rovira, Alfredo Rodríguez-Antigüedad

**Affiliations:** ^1^Departamento de Farmacología, Facultad de Medicina, Universidad de Málaga, 29016 Málaga, España; ^2^Instituto de Investigación Biomédica de Málaga (IBIMA), Hospital Universitario Regional de Málaga, Universidad de Málaga, 29016 Málaga, España; ^3^Centro de Esclerosis Múltiple de Cataluña y Departamento de Neurología, Hospital Universitario Vall d’Hebron, 08035 Barcelona, España; ^4^Universitat Autònoma de Barcelona, 08193 Bellaterra, España; ^5^Servicio de Neurología, Complejo Asistencial Universitario de León, 24008 León, España; ^6^Servicio de Neurología, Hospital Reina Sofía, 14004 Córdoba, España; ^7^Servicio de Neurología, Hospital Universitario de Getafe, 28905 Getafe, España; ^8^Unidad de Esclerosis Múltiple, Servicio de Neurología, Hospital Regional Universitario de Málaga, 29010 Málaga, España; ^9^Servicio de Neurología, Hospital Universitario Quirónsalud, 28223 Pozuelo de Alarcón, España; ^10^Universitat de Lleida, 25198 Lleida, España; ^11^Hospital Universitari Arnau de Vilanova, Institut de Recerca Biomèdica de Lleida (IRBLleida), 25198 Lleida, España; ^12^Servicio de Neurología, Hospital Universitario Son Espases, 07120 Palma de Mallorca, España; ^13^Servicio de Neurología, Complejo Asistencial de Ávila, 05071 Ávila, España; ^14^Servicio de Neurología, Hospital Universitario Donostia, Grupo de Neuroinmunología, IIS Biogipuzkoa, 20014 Donostia, España; ^15^CSUR de Esclerosis Múltiple, Hospital Ramón y Cajal, 28034 Madrid, España; ^16^Servicio de Neurología, Hospital Universitario Virgen Macarena, 41009 Sevilla, España; ^17^Servicio de Neurología, Hospital Universitario Doctor Peset, 46017 Valencia, España; ^18^Servicio de Neurología, Hospital Universitario Central de Asturias, 33011 Oviedo, España; ^19^Unidad de Neuroinmunología y Esclerosis Múltiple, Hospital Clínic de Barcelona e IDIBAPS, 08036 Barcelona, España; ^20^CSUR Esclerosis Múltiple, Unidad de Neuroinmunología Clínica, Servicio de Neurología, Hospital Clínico Universitario Virgen de la Arrixaca (IMIB-Arrixaca), 30120 El Palmar, España; ^21^Cátedra de Neuroinmunología Clínica y Esclerosis Múltiple, Universidad Católica San Antonio (UCAM), 30107 Guadalupe, España; ^22^Servicio de Neurología, Hospital Universitario de la Princesa, 28006 Madrid, España; ^23^Servicio de Neurología, Complejo Hospitalario Universitario Moisès Broggi, 08970 Barcelona, España; ^24^Servicio de Neurología, Hospital Clínico San Carlos, Instituto de Investigación Sanitaria San Carlos (IdISSC), 28040 Madrid, España; ^25^Departamento de Medicina, Facultad de Medicina, Universidad Complutense de Madrid (UCM), 28040 Madrid, España; ^26^Servicio de Neurología, Instituto de Investigación Sanitaria de Santiago de Compostela (IDIS), 15706 Santiago de Compostela, España; ^27^Hospital Universitari de Bellvitge-Institut d'Investigació Biomèdica de Bellvitge (IDIBELL), 08908 L’Hospitalet de Llobregat, España; ^28^Sección de Neuroradiología, Departamento de Radiología, Hospital Universitari Vall d'Hebron, 08035 Barcelona, España; ^29^Universitat Autònoma de Barcelona, 08193 Barcelona, España; ^30^Servicio de Neurología, Hospital Universitario Cruces, 48903 Barakaldo, España

**Keywords:** ECTRIMS, esclerosis múltiple, post-ECTRIMS, ECTRIMS, multiple sclerosis, post-ECTRIMS

## Abstract

La XVIII edición de la reunión post-ECTRIMS (*European Committee for Treatment and Research in Multiple Sclerosis*) se celebró los días 17 y 18 de octubre de 2025 en Madrid, congregando a neurólogos españoles especializados en esclerosis múltiple (EM), quienes presentaron los avances más relevantes discutidos en el congreso ECTRIMS, celebrado días antes en Barcelona. El objetivo de la reunión fue resumir las novedades sobre patogenia, nuevos criterios diagnósticos y biomarcadores de la EM. Este artículo corresponde al primero de una serie de dos artículos (I y II). En él se revisan datos recientes sobre el tráfico celular hacia el sistema nervioso central, destacando la heterogeneidad funcional del endotelio cerebral, los mecanismos moleculares que regulan la infiltración leucocitaria y el papel diferencial de macrófagos y microglía residente en el daño tisular y la inflamación crónica. Se presenta la evidencia que respalda el papel del virus de Epstein-Barr como factor de riesgo necesario en la EM, junto con las implicaciones terapéuticas y preventivas derivadas de los estudios con terapias antivirales, celulares y vacunas. En el ámbito diagnóstico, se abordan los nuevos criterios de los trastorno del espectro de la neuromielitis óptica, con especial atención a la heterogeneidad molecular de las formas doble seronegativas, así como la actualización de las guías de resonancia magnética (RM) tras la revisión de los criterios de McDonald 2024. Se destacan los avances en neuroimagen de la inflamación crónica activa, la imagen medular cuantitativa y el concepto de reserva de la médula espinal. Asimismo, se revisa el creciente papel de la inteligencia artificial aplicada a la RM para la predicción precoz de progresión y respuesta al tratamiento. Por último, se presentan avances en mecanismos de remielinización y en biomarcadores, subrayando el valor de enfoques multimodales y longitudinales para la estratificación pronóstica y la monitorización terapéutica. En conjunto, los datos revisados reflejan una evolución hacia una comprensión más integrada y predictiva de la EM, con implicaciones directas para el diagnóstico precoz, la personalización del tratamiento y el diseño de futuros ensayos clínicos.

## Abstract (en)

The 18th edition of the post-European Committee for Treatment and Research in Multiple Sclerosis (ECTRIMS) meeting was held on 17–18 October 2025 in Madrid, bringing together Spanish neurologists specialising in multiple sclerosis (MS), who presented the most relevant advances discussed at the ECTRIMS congress held a few days earlier in Barcelona. The aim of the meeting was to summarise the latest developments in the pathogenesis, new diagnostic criteria and biomarkers of MS. This article is the first of a two-part series (I and II) that reviews recent data on cell trafficking into the central nervous system, highlighting the functional heterogeneity of the brain endothelium, the molecular mechanisms regulating leukocyte infiltration and the differential role of macrophages and resident microglia in tissue damage and chronic inflammation. The evidence supporting the role of Epstein-Barr virus as a necessary risk factor in MS is presented, together with the therapeutic and preventive implications from studies of antiviral therapies, cell-based therapies and vaccines. Regarding diagnosis, the new criteria for neuromyelitis optica spectrum disorders are addressed, with particular attention to the molecular heterogeneity of double-seronegative forms, as well as the update of the magnetic resonance imaging (MRI) guidelines following the 2024 revision of the McDonald criteria. Advances in the neuroimaging of chronic active inflammation, quantitative spinal cord imaging and the concept of spinal cord reserve are highlighted. The growing role of artificial intelligence applied to MRI for the early prediction of progression and treatment response is also reviewed. Finally, advances in remyelination mechanisms and in biomarkers are presented, underscoring the value of multimodal and longitudinal approaches for prognostic stratification and treatment monitoring. Overall, the data reviewed reflect a change into a more integrated and predictive understanding of MS, with direct implications for early diagnosis, treatment personalisation and the design of future clinical trials.

## 1. Introducción

Los días 17 y 18 de octubre de 2025 tuvo lugar la XVIII edición de la reunión post-ECTRIMS (*European Committee for Treatment and Research in Multiple Sclerosis*). Como cada año, varios neurólogos expertos en esclerosis múltiple (EM) en España presentaron un resumen de las principales novedades del congreso ECTRIMS celebrado en Barcelona los días 24–26 de septiembre. Los resúmenes de dichas ponencias se presentan en dos artículos (I y II).

En este primer artículo (I), se incluyen las novedades en biología y fisiopatología de la enfermedad, con especial atención al tráfico celular y a las células inmunes en el sistema nervioso central (SNC), así como al papel del virus de Epstein-Barr en la EM. También se abordan los nuevos criterios diagnósticos del trastorno del espectro de la neuromielitis óptica (NMOSD, *neuromyelitis optica spectrum disorder*) y la actualización de las guías de resonancia magnética (RM) tras la revisión de los criterios diagnósticos de EM [1]. También se presentan los avances en imagen aplicados a la inflamación crónica y a la médula espinal, junto con las nuevas perspectivas que ofrece la inteligencia artificial (IA) en el ámbito de la neuroimagen. Por último, se revisan los mecanismos de remielinización, los biomarcadores en fluidos corporales y las herramientas estadísticas emergentes para la medición y predicción de los resultados clínicos.

La selección de ponencias se realizó siguiendo un enfoque temático coherente con el utilizado en ediciones previas del Post-ECTRIMS, con el objetivo de garantizar una cobertura equilibrada y dar prioridad a las líneas de investigación con un posible mayor impacto en la práctica clínica.

## 2. Tráfico Celular y Células Inmunes en el Sistema Nervioso Central

### 2.1 Papel del Endotelio Cerebral en la EM

En condiciones fisiológicas, el SNC está protegido por la barrera hematoencefálica (BHE), formada por células endoteliales que impiden el paso de células inmunes. En la EM, esta barrera pierde su integridad y permite la entrada de linfocitos T CD4 y CD8 (CD, grupo de diferenciación [*cluster of differentiation*]), macrófagos, células dendríticas, y linfocitos B y T reguladores hacia el parénquima, donde contribuyen al daño de oligodendrocitos y neuronas [2].

Estudios recientes en modelos animales de encefalomielitis autoinmunitaria experimental (EAE) han mostrado que el endotelio cerebral presenta heterogeneidad transcriptómica a lo largo del árbol vascular, con un fenotipo inflamatorio caracterizado por la expresión de moléculas de adhesión (VCAM-1), receptores quimiotácticos (ACKR1) y genes de respuesta inflamatoria como *LCN2*. Esta diversidad endotelial parece modular la localización y extensión de las lesiones inflamatorias [3]. Datos transcriptómicos en humanos confirman diferencias regionales entre las células endoteliales del tronco encefálico, la corteza, el cerebelo y la sustancia blanca, en tejido sano [4].

### 2.2 Tráfico de Leucocitos Hacia el Sistema Nervioso Central

Se han identificado 18 genes que facilitan la infiltración de células T al SNC (siendo *Itga4*, *Fermt3* y *Hsp90b1* los genes principales). La infiltración de leucocitos al SNC depende de dos mecanismos. Un mecanismo de adhesión que incluye la integrina α4 y su proteína asociada intracelular, kindlina-3 (codificada por *Fermt3*), junto con la chaperona HSP90B1. Estas moléculas participan en la activación de las integrinas y en la infiltración de linfocitos T autoreactivos [5].

El segundo mecanismo es la polarización funcional de los macrófagos infiltrantes. Los macrófagos infiltrantes adoptan distintos fenotipos según las señales del microambiente [6]. El interferón-γ (IFN-γ) favorece un fenotipo proinflamatorio con alta actividad oxidativa, mientras que el factor transformador de crecimiento β (TGF-β*, transforming growth factor-β*) promueve un estado antiinflamatorio y reparador, requerido para la eliminación de lípidos de mielina y la prevención de un fenotipo “espumoso” disfuncional. En la EAE, la ausencia de señalización por TGF-β conduce a una acumulación desorganizada de macrófagos dentro de la lesión [7]. Los mismos patrones de polarización inducidos por IFN-γ y TGF-β se han confirmado en la EM [8].

### 2.3 Interacciones de las Células Inmunes y los Mediadores Inmunitarios en el Sistema Nervioso Central

Las interacciones entre las células inmunes y las del parénquima cerebral—astrocitos, microglía, neuronas y oligodendrocitos—son determinantes en la patogenia de la EM y difieren de las observadas en otras enfermedades desmielinizantes. Además, cabe destacar que la microglía puede tener una función protectora o lesional según fase y nicho. En la EAE, el daño tisular y la neurodegeneración son promovidos principalmente por macrófagos derivados de monocitos, mientras que en la EM, la lesión activa inicial y la expansión de las lesiones crónicas están impulsadas por la activación persistente de la microglía proinflamatoria [9].

Durante la inflamación, en la EAE los monocitos inflamatorios Ly6C^hi^ (el equivalente humano de CD14++/CD16–) que penetran en el SNC maduran a macrófagos tisulares mediante una transición regulada por citocinas inflamatorias (transición monocito-fagocito). Estos macrófagos expresan receptor tirosina-cinasa MER (MerTK, *MER tyrosine kinase*) y participan en la eliminación de detritos y la resolución de la inflamación. En cambio, la microglía residente mantiene su identidad y una función moduladora más que patogénica.

Entre las citocinas implicadas, el factor estimulante de colonias de granulocitos-macrófagos (GM-CSF, *granulocyte–macrophage colony-stimulating factor*) se encuentra desregulado en la EM. Su sobreexpresión por linfocitos T CD4 es suficiente para inducir neuroinflamación grave, caracterizada por infiltración de monocitos inflamatorios que fagocitan mielina en tronco encefálico, cerebelo y meninges. Este hallazgo refuerza que las citocinas desreguladas pueden generar inflamaciones órgano-específicas: interleucina (IL)-17 en piel, factor de necrosis tumoral (TNF, *tumor necrosis factor*) en articulaciones y GM-CSF en el SNC. Estos datos indican que los fagocitos infiltrantes, más numerosos que las células T, son los principales ejecutores del daño inmunitario, mientras que la microglía residente probablemente ejerce una función protectora y contrarresta el daño causado por los macrófagos derivados de monocitos.

## 3. Virus de Epstein-Barr en la EM

### 3.1 Estudios Epidemiológicos

La infección por el virus de Epstein-Barr (VEB) se ha consolidado como un factor de riesgo con evidencia consistente con un papel causal mayor para el desarrollo de la EM. Los datos epidemiológicos muestran que prácticamente todos los pacientes con EM son seropositivos frente al VEB. En el estudio de Bjornevik ya se mostró que el riesgo de EM aumentó 32 veces tras la infección por el VEB, pero no por la infección por otros virus, incluido el citomegalovirus [10]. Los neurofilamentos de cadena ligera (NfL, *neurofilament light chain*) aumentaron únicamente después de la seroconversión al VEB. Estos hallazgos no pueden explicarse por ningún factor de riesgo conocido para la EM y sugieren que el VEB tiene un papel principal y necesario, aunque no suficiente, en la enfermedad [10].

### 3.2 Control Inmunitario de la Infección por Virus de Epstein-Barr

**Fig. 1.**
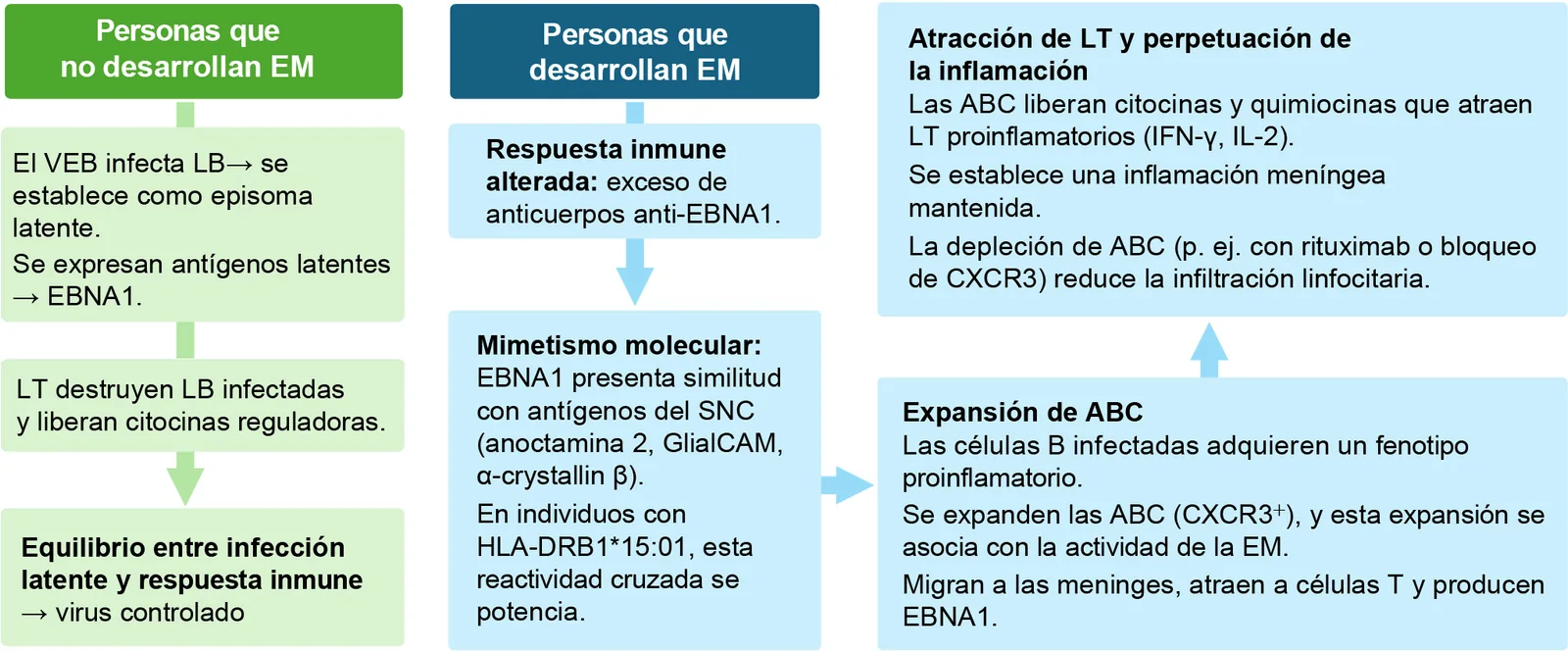
**Control inmunitario del virus de Epstein-Barr**. Abreviaturas: ABC, células B atípicas (*atypical B cells*); CXCR3, receptor de quimiocinas C-X-C tipo 3 (*C-X-C chemokine receptor 3*); EBNA1, antígeno nuclear 1 del virus de Epstein–Barr (*Epstein–Barr nuclear antigen 1*); EM, esclerosis múltiple; HLA, antígeno leucocitario humano (*human leukocyte antigen*); IFN-γ, interferón-γ (*interferon gamma*); IL-2, interleucina 2 (*interleukin-2*); LB, linfocitos B; LT, linfocitos T; SNC, sistema nervioso central; VEB, virus de Epstein-Barr.

El VEB ha desarrollado mecanismos de evasión inmunitaria que le permiten persistir en las células B memoria, su principal reservorio. En condiciones normales, las células T citotóxicas controlan la infección, manteniendo el virus en un estado de latencia. En la EM, este equilibrio se rompe y los pacientes muestran una respuesta alterada frente al virus (ver Fig. 1).

### 3.3 Diana para el Tratamiento y la Prevención de la EM

Un estudio de cohorte concluyó que existe un menor riesgo de EM tanto en personas con virus de inmunodeficiencia humana (VIH) como en aquellas expuestas a tratamiento antirretroviral [11]. Sin embargo, aún no disponemos de una terapia dirigida al VEB capaz de modificar el curso de la EM. El ensayo EMBOLD no logró demostrar la eficacia de ATA188, una terapia celular alogénica basada en linfocitos T específicos del VEB, frente a placebo en pacientes con EM progresiva. De especial interés son los estudios con tenofovir alafenamida fumarato (TAF), que inhibe la replicación lítica del VEB, cuyos resultados se esperan pronto [12]. En prevención, las vacunas gp350 reducen la mononucleosis infecciosa pero no previenen la infección por VEB ni el riesgo de EM [12].

## 4. Nuevos Criterios Diagnósticos de los Trastornos del Espectro de la Neuromielitis Óptica

### 4.1 Características Moleculares de la Enfermedad Doble Seronegativa

La caracterización molecular del NMOSD doble seronegativo (DS-NMOSD) parece ser más similar a la de enfermedad asociada a anticuerpos contra la glicoproteína de oligodendrocitos de mielina (MOGAD, *myelin oligodendrocyte glycoprotein antibody-associated disease*) o EM que a acuaporina-4 (AQP4, *aquaporin-4*)+NMOSD [13]. Los pacientes con AQP4+NMOSD presentan mayores niveles de proteína ácida fibrilar glial (GFAP, *glial fibrillary acidic protein*), NfL, tau y ubiquitin C-terminal hydrolase-L1 (UCH-L1) en líquido cefalorraquídeo, y de tau y UCHL-L1 en suero [13,14]. Los análisis proteómicos refuerzan esta diferenciación; decenas de proteínas aparecen diferencialmente expresadas entre AQP4+NMOSD, MOGAD y DS-NMOSD [15]. Estos hallazgos sugieren que la DS-NMOSD no constituye un fenotipo homogéneo, sino una entidad molecularmente diversa que exige un abordaje diagnóstico refinado en los nuevos criterios de NMOSD.

### 4.2 Criterios Diagnósticos para la NMOSD

La Tabla 1 resume las novedades sobre los criterios diagnósticos para la NMOSD del panel internacional IPND (*International Panel for Neuromyelitis Optica Diagnosis*) 2025 [16,17]. Las principales novedades son la incorporación del cerebelo como región típica, la prioridad del ensayo celular vivo (*live cell-based assay*, LCBA), y la distinción de enfermedad y síndrome en seronegativos.

**Tabla 1.** **Criterios diagnósticos para la NMOSD**.

Área	Novedades
Serología AQP4-IgG	• Suero como muestra recomendada
	• LCBA es el *gold standard*
	• FCBA aceptada si positiva
	• ELISA no recomendado
	• Repetir muestra si resultado negativo con técnica subóptima o en condiciones no ideales
Patrones de RM para AQP4-IgG+	• Cerebelo: región dentada
	• Tronco encefálico: 4º ventrículo
	• Cerebro: 3º ventrículo; realce ependimario lineal
	• Médula: LETM con *bright spotty lesions*
Algoritmo diagnóstico	Cuadro clínico compatible + serología:
	• LCBA
	– positiva → NMOSD
	– negativa → seronegativo confirmado
	• FCBA o ELISA:
	– positiva → NMOSD
	– negativa → seronegativo probable
	• No evaluados.
Clasificación	Se introduce una clasificación dual:
	• Enfermedad (seropositiva): MOGAD y NMOSD con AQP4-IgG+.
	• Síndromes (seronegativa):
	– síndrome NMO clásico (criterios de 2015)
	– fenotipos clínicos descriptivos
Factores clínicos que generan falsos negativos	• Corticoides y plasmaféresis reducen transitoriamente los títulos de AQP4-IgG
	• Inmunosupresores inespecíficos y anticuerpos monoclonales pueden inducir “seroreversión”

Abreviaturas: AQP4, acuaporina-4 (*aquaporin-4*); ELISA, ensayo inmunoenzimático (*enzyme-linked immunosorbent assay*); FCBA, ensayo celular fijo (*fixed cell-based assay*); LCBA, ensayo celular vivo (*live cell-based assay*); LETM, mielitis transversa longitudinalmente extensa (*longitudinally extensive transverse myelitis*); NMOSD, trastorno del espectro de la neuromielitis óptica (*neuromyelitis optica spectrum disorder*); MOGAD, enfermedad asociada a anticuerpos contra la glicoproteína de oligodendrocitos de mielina (*myelin oligodendrocyte glycoprotein antibody-associated disease*); RM, resonancia magnética; NMO, neuromielitis óptica. Las flechas (→) indican la clasificación diagnóstica resultante de cada resultado serológico.

## 5. Actualización de las Guías de Resonancia Magnética para la Revisión de 2024 de los Criterios Diagnósticos de EM (MAGNIMS, CMSC y NAIMS)

La actualización de las guías MAGNIMS-CMSC-NAIMS (MAGNIMS, *Magnetic Resonance Imaging in Multiple Sclerosis;* CMSC, *Consortium of Multiple Sclerosis Centers*; NAIMS, *North American Imaging in Multiple Sclerosis*) refuerza la necesidad de incorporar de forma sistemática la evaluación del nervio óptico en el proceso diagnóstico de la EM, integrando herramientas estructurales y funcionales. La RM orbitaria dedicada, con secuencias ponderadas en T2 con supresión grasa (T2-FS, *T2 fat-suppressed*, o STIR, *short tau inversion recovery*), un grosor de corte 2–3 mm y cobertura hasta el quiasma, permite detectar hiperintensidades en T2 y lesiones compatibles con neuritis óptica, aunque su sensibilidad para afectación asintomática sigue siendo limitada. Secuencias de RM no dedicadas que abarcan además de la vía óptica, el parénquima cerebral, como la secuencia tridimensional de doble inversión-recuperación (3D DIR, *three-dimensional double inversion recovery*) obtenidas con vóxeles isotrópicos de ≤1 mm^3^, han demostrado una elevada sensibilidad en la detección no solo sintomáticas, sino también asintomáticas de la vía óptica anterior [18].

La tomografía de coherencia óptica (OCT, *optical coherence tomography*) aporta medidas estructurales, donde la asimetría interocular (≥5 µm para la capa de fibras nerviosas peripapilares de la retina [pRNFL, *peripapillary retinal nerve fiber layer*] o ≥4 µm para la capa de células ganglionares e interna plexiforme [GCIPL, *ganglion cell–inner plexiform layer*]) orienta a daño axonal previo; y los potenciales evocados visuales (PEV) complementan la evaluación funcional mediante la identificación de latencias P100 prolongadas. Se propone, además, un algoritmo para integrar la afectación óptica en la toma de decisiones diagnósticas, especialmente en pacientes con síndrome clínico aislado (CIS, *clinically isolated syndrome*), permitiendo reclasificar casos que no cumplían los criterios de 2017 [19] y mejorar la identificación precoz de la diseminación en el espacio según los criterios de 2024 [1,20].

La utilidad del signo de la vena central (CVS, *central vein sign*) y las lesiones con borde paramagnético (PRLs, *paramagnetic rim lesions*) es más controvertida, ya que requieren el uso de secuencias optimizadas sensibles a la susceptibilidad magnética para identificarlas con precisión y experiencia para interpretarlas. En el caso del CVS, aunque el método basado en evaluar >40% de lesiones venocéntricas es robusto, resulta laborioso y con variabilidad interobservador. Por ello, se han propuesto métodos simplificados como la regla del 6, incorporada en los criterios de 2024, que considera positivo al paciente si al menos seis lesiones son CVS+. En personas con <10 lesiones de sustancia blanca, se aplica que el número de lesiones CVS+ debe ser mayor que el de lesiones CVS–. Respecto a las PRLs, su detección exige técnicas sensibles a susceptibilidad magnética (SWI [*susceptibility-weighted imaging*], SWIp [SWI with phase], SWAN [*susceptibility-weighted angiography*] con imagen de fase o QSM [*quantitative susceptibility mapping*]), y, aunque no son un marcador específico de formas progresivas, sí reflejan biología inflamatoria crónica y podrían contribuir a caracterizar fenotipos con mayor riesgo de progresión [1,20]. En un estudio realizado en el Vall d’Hebron, la aplicación de los criterios de McDonald 2024 incrementó la proporción de diagnósticos de EM del 50% al 72%, lo que supone un aumento relativo del 42,7% respecto a los criterios de 2017 [21].

La Tabla 2 muestra el protocolo estandarizado de RM para el diagnóstico de EM, detallando los parámetros y las secuencias recomendadas para cerebro, médula espinal y nervio óptico según las guías MAGNIMS-CMSC-NAIMS 2024 [20].

**Tabla 2.** **Protocolo estandarizado de RM para el diagnóstico de EM según las guías MAGNIMS-CMSC-NAIMS 2024**.

Parámetro	Encéfalo	Médula espinal	Nervio óptico
Intensidad de campo	≥1,5 T	≥1,5 T	≥1,5 T
Adquisición	3D preferido	2D o 3D	2D o 3D
Grosor de corte	3D: 1 mm iso; 2D: <3 mm, sin separación	Sagital ≤3 mm; sin separación; Axial ≤5 mm, sin separación	2–3 mm, sin separación
Resolución en plano	≤1 mm × 1 mm	≤1 mm × 1 mm	≤1 mm × 1 mm
Cobertura	Encéfalo completo	Médula completa	Nervios ópticos y quiasma
Orientación axial	Plano subcalloso o AC-PC	Perpendicular al eje sagital	Alinear con nervio óptico y quiasma
Secuencias principales	3D T2 FLAIR; Axial T2w; 3D T2* GRE/SWI para CVS; Post-GBCA axial T1w	2 secuencias sagitales (T2, PD, STIR, PSIR, MPRAGE); Axial 2D T2w; Post-GBCA sagital T1w	Coronal FS T2w o STIR; Post-GBCA coronal FS T1w
Secuencias adicionales	3D T2* GRE (SWI) para PRL; DWI; DIR/PSIR; 3D T1w de alta resolución	Post-GBCA axial T1w a través de lesiones	Axial FS T2/STIR; Post-GBCA T1w; 3D DIR

Abreviaturas: 2D, bidimensional; 3D, tridimensional; AC-PC, línea comisura anterior–comisura posterior; CVS, signo de la vena central (*central vein sign*); DIR, secuencia de doble inversión-recuperación (*double inversion recovery*); DWI, difusión por RM (*diffusion-weighted imaging*); FLAIR, recuperación por inversión con atenuación de líquido (*fluid-attenuated inversion recovery*); FS, supresión de grasa (*fat-suppressed*); GBCA, agente de contraste basado en gadolinio (*gadolinium-based contrast agent*); GRE, eco de gradiente (*gradient echo)*; iso, isotrópico; mm, milímetros; MAGNIMS-CMSC-NAIMS (MAGNIMS, *Magnetic Resonance Imaging in Multiple Sclerosis*; CMSC, *Consortium of Multiple Sclerosis Centers*; NAIMS, *North American Imaging in Multiple Sclerosis*); MPRAGE, eco de gradiente rápido con preparación por magnetización (*magnetization-prepared rapid gradient echo*); PD, densidad protónica (*proton density*); PRLs, lesiones paramagnéticas en anillo (*paramagnetic rim lesions*); PSIR, inversión-recuperación con sensibilidad de fase (*phase-sensitive inversion recovery*); STIR, recuperación por inversión con tau corta (*short tau inversion recovery*); SWI, imagen ponderada por susceptibilidad (*susceptibility-weighted imaging*); T, tesla; T1w, ponderada en T1 (*T1 weighted*); T2w, ponderada en T2 (*T2 weighted*); T2*, T2 estrella.

## 6. Imagen de la Inflamación Crónica

**Fig. 2.**
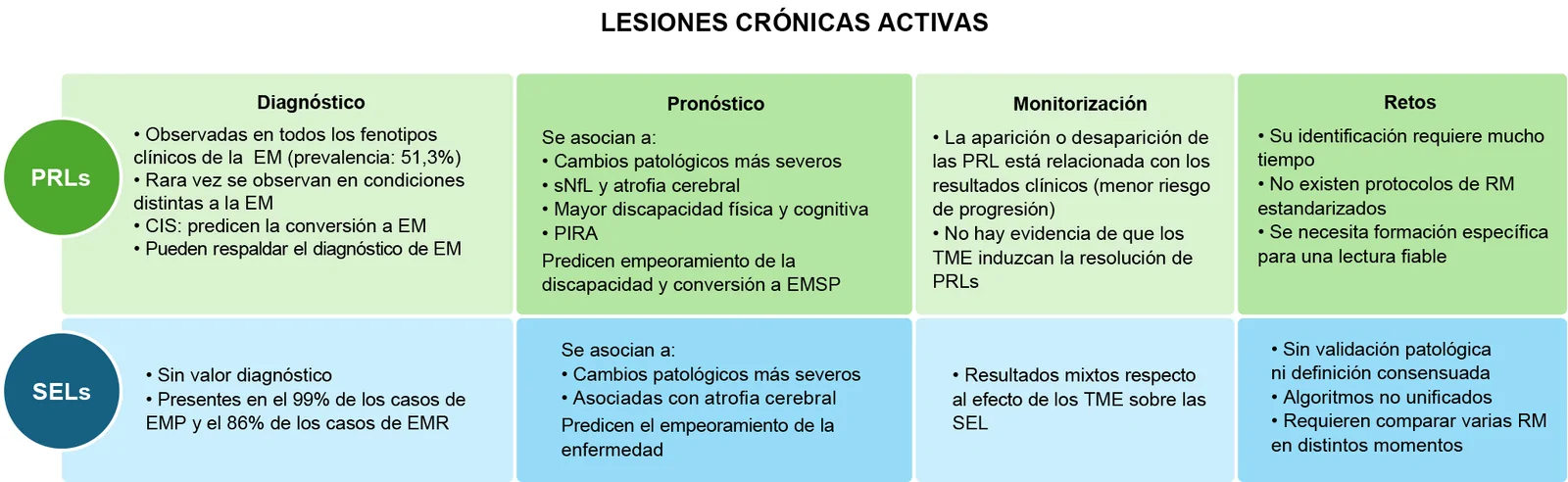
**Lesiones crónicas activas**. Abreviaturas: PIRA, progresión independiente de brotes (*progression independent of relapse activity*); sNfL, neurofilamentos de cadena ligera en suero (*serum neurofilament light chain*); SELs, lesiones de expansión lenta (*slowly expanding lesions*); TME, tratamiento modificador de la enfermedad; EMSP, esclerosis múltiple secundaria progresiva; EMP, esclerosis múltiple progresiva; EMR, esclerosis múltiple recurrente.

Los biomarcadores de RM permiten identificar lesiones con actividad inflamatoria crónica, como las PRLs o las lesiones de expansión lenta (SELs, *slowly expanding lesions*), mientras que la tomografía por emisión de positrones (PET, *positron emission tomography*) detecta las lesiones positivas para proteína translocadora (TSPO). Su detección ayuda a afinar el diagnóstico diferencial, anticipar el pronóstico y personalizar las decisiones terapéuticas [22]. La Fig. 2 muestra el valor diagnóstico, pronóstico y de monitorización de las PRLs y las SELs. Las SELs son entre 2–8 veces más frecuentes que las PRLs, y menos del 20% de las SELs están colocalizadas con PRLs, lo que refleja que ambos biomarcadores detectan aspectos diferentes de inflamación crónica, siendo estas lesiones las que expresan una mayor gravedad de la enfermedad [23].

Tanto los estudios de imagen con RM postmortem como los estudios de PET proporcionan información sobre la patología de la EM [23,24]. La PET TSPO muestra inflamación crónica no captada por la RM. En los estudios presentados, la activación microglial fue extensa en formas progresivas y se localizó en los bordes de las lesiones activas crónicas, mientras que los astrocitos reactivos mostraron un patrón complementario. La PET permitió diferenciar progresión lenta frente a rápida, apuntando a las *broad rim lesions* como un posible nuevo marcador asociado a progresión rápida [25], y mostró que la microglía permaneció activada en la EM secundaria progresiva (EMSP) sin tratamiento, mientras que algunos tratamiento modificador de la enfermedad (TME), como natalizumab, se asoció con una reducción en el volumen de microglía activa. Estos datos refuerzan el potencial de la PET como herramienta para monitorizar inflamación activa de forma temprana en ensayos clínicos.

## 7. Imagen de la Médula Espinal

La afectación medular es frecuente en la EM, con lesiones presentes en >90% de los pacientes con EM clínicamente definida y con implicaciones diagnósticas relevantes en la revisión de los criterios McDonald 2024 [1]. Más allá de las lesiones focales, diversas medidas cuantitativas aportan información sobre la progresión; el área transversal del segmento cervical (SCA, *spinal cord area*) muestra una reducción del 9,6% en EM frente a controles sanos y una tasa de cambio anual del 1,78%, mostrando una fuerte asociación con la discapacidad concomitante y futura [26].

Las medidas microestructurales del estado del tejido complementan esta información. Entre ellas, el ratio de transferencia de magnetización (MTR, *magnetization transfer ratio*) y la imagen por tensor de difusión (DTI, *diffusion tensor imaging*) presentan una asociación moderada con la discapacidad presente y futura. Hay evidencia del impacto de estas medidas a nivel individual en la progresión, si bien persisten dificultades técnicas y variabilidad intercentro en la evaluación de estas medidas [26].

El concepto de reserva medular (*spinal cord reserve*) amplía la visión de la médula, integrándola en el marco de la “reserva del SNC” junto a las dimensiones física, cognitiva y cerebral [27]. Estudios recientes muestran que el volumen basal del área del canal cervical, un marcador indirecto de la reserva medular, correlaciona con la escala ampliada del estado de discapacidad (EDSS, *Expanded Disability Status Scale*) y predice la discapacidad a cinco años, incluso tras ajustar por la fracción de parénquima medular (área medular dividida por el área del canal cervical), carga lesional, edad y sexo [28,29]. Los resultados de estos estudios y de otros estudios recientes sobre imagen de médula espinal se muestran en la Tabla 3 (Ref. [28,29,30,31,32,33]).

**Tabla 3.** **Estudios de imagen de médula espinal en EM**.

Referencia	Objetivo	N	Resultados
Ajdinaj et al. [29]	Evaluar si el CCaA, medido en el primer evento desmielinizante, se asocia con la acumulación de discapacidad en EM.	121 pacientes con CIS.	Aunque el CCaA no predijo la EDSS al inicio, su efecto sobre la acumulación de discapacidad aumentó con el tiempo, con una interacción CCaA–tiempo significativa en C3/C4.
Mongay-Ochoa et al. [28]	Medir si el CCaA puede utilizarse como marcador indirecto de la reserva medular en una cohorte multicéntrica de personas con EM.	177 C,	Los pacientes con EMP mostraron un CCaA menor que los C y los pacientes con EMR tanto en C2/C3 como en C3/C4. No hubo diferencias entre C y EMR.
		289 pacientes con EMR,	
		139 pacientes con EMP.	
			En C3/C4, un CCaA menor se asoció con una EDSS basal más alta, y los pacientes que empeoraron clínicamente a 5 años presentaban un CCaA basal más reducido que aquellos clínicamente estables.
Tsagkas [30]	Identificar el daño medular mediante RM post mortem de ultra-alta resolución (9,4 T) combinada con histología.	22 especímenes medulares post mortem (18 pacientes con EM; 2 ELA; 2 C enfermedad neurológica no inflamatoria) + 9 casos *in vivo.*	Predominio lateral de las lesiones, seguido de regiones posterior, central y anterior. Lesiones de SG pura (6%), predominio de SG (36%), mixtas SG/SB (33%), predominio de SB (22%) y SB pura (4%).
			La RM clínica (1,5–3 T) detecta pocas lesiones, especialmente en niveles torácicos y lumbares; las más visibles son las que afectan simultáneamente SG + SB.
			La mayoría de las lesiones muestran CVS o múltiples venas. Se observaron PRL medulares, que en la histología corresponden a lesiones mixtas activas-inactivas con desmielinización continua.
Eunyoung-Lee [31]	Evaluar si el MTR en médula cervical en fases iniciales de la EM se asocia con el riesgo de progresión de la discapacidad a 5 años.	237 pacientes con EM.	Niveles más elevados de MTR se asociaron con menor riesgo de progresión de la EDSS, ajustado por edad, fenotipo, EDSS basal y centro.
			La asociación se observó tanto en medidas globales de la médula cervical como en tramos específicos de SB (columnas dorsales, funículos laterales, tractos corticospinales).
			Los pacientes con MTR más bajo mostraron mayor riesgo de progresión.
Hong et al. [32]	Comparar la detección de actividad radiológica usando solo RM cerebral versus RM cerebral + medular, e identificar factores asociados a nuevas lesiones medulares.	68 pacientes con EM.	Media de 3 lesiones medulares por paciente (rango 2–6). Solo 7% recibía TME de alta eficacia.
			En el 46% de los intervalos sin brotes se identificó actividad radiológica. En estos intervalos se detectaron nuevas lesiones medulares aisladas en el 2%.
			En los intervalos con actividad clínica se identificaron nuevas lesiones medulares aisladas en el 10%.
			Mayor riesgo de nuevas lesiones medulares cuanto mayor número de lesiones cerebrales basales y nuevas (OR 1,17). El número basal de lesiones medulares no se asoció a riesgo de nuevas lesiones (*p* = 0,6).
			Los hallazgos sugieren que en ciertos escenarios podría ser suficiente monitorizar únicamente con RM cerebral, (salvo en casos con síntomas medulares, progresión, discordancias clínico-RM, etc.).
Jain [33]	Modelar trayectorias de atrofia de la médula cervical (nMUCCA) en C y establecer patrones de cambios en pacientes con EM.	480 C y 1.295 pacientes con EM	En C: patrón no lineal con incremento hasta los 30 años y reducción progresiva a partir de los 50.
			En EM: reducción significativa del nMUCCA hasta los 50 años, con estabilización posterior.
			Asociación negativa entre duración de la enfermedad y nMUCCA; en inicio pediátrico, la reducción fue más marcada.
			Correlación moderada entre EDSS y nMUCCA z-score (rho = –0,35, *p * < 0,001).

Abreviaturas: C, controles; CCaA, área del canal cervical (*cervical canal area*); CIS, síndrome clínico aislado (*clinically isolated syndrome*); EDSS, escala ampliada del estado de discapacidad (*Expanded Disability Status Scale*); ELA, esclerosis lateral amiotrófica; MTR, ratio de transferencia de magnetización (*magnetization transfer ratio*); nMUCCA, área cervical superior media normalizada (*normalized mean upper cervical cord área*); OR, razón de probabilidades (*odds ratio*); SB, sustancia blanca; SG, sustancia gris.

## 8. Inteligencia Artificial y Resonancia Magnética

El número de estudios que utiliza la IA aplicada a la RM para predecir resultados clínicos está aumentando considerablemente [34]. Los modelos de IA han obtenido buenos resultados en la estimación de respuesta al tratamiento y en la identificación de fenotipos biológicos, con un desarrollo especialmente prometedor en monitorización [34,35]. Sin embargo, las predicciones clínicas aún muestran precisión moderada y escasa validación externa, lo que limita su traslación inmediata a la práctica [34].

Respecto a los estudios dirigidos a predecir la progresión de la discapacidad, destaca el de Coll et al. [36], que describe un modelo de *deep learning* capaz de predecir la progresión independiente de brotes (PIRA, *progression independent of relapse activity*) desde el primer episodio desmielinizante usando únicamente RM rutinaria. El modelo alcanzó una concordancia elevada (C-índice 0,72), mejoró las predicciones clásicas ajustadas por edad y permitió identificar regiones cerebrales que predicen PIRA, como la corteza frontoparietal, lo que sugiere su utilidad potencial para estratificar el riesgo de forma temprana en la práctica clínica, si bien estos resultados precisan validación externa.

En cuanto al uso de la IA para predecir la respuesta al tratamiento y optimizar el diseño de ensayos clínicos, destacaron dos estudios. Falet et al. [37] entrenaron una red neuronal multicapa con datos de tres ensayos fase III (n = 3820) de anti-CD20, laquinimod e interferón beta, incorporando variables demográficas, EDSS y volumen lesional basal. El modelo estimó el efecto individual del tratamiento sobre la progresión de la discapacidad y permitió clasificar a los pacientes en respondedores y no respondedores según umbrales de enriquecimiento predictivo. Además, vieron que, a mayor grado de enriquecimiento predictivo basado en IA, menor es el tamaño muestral necesario para detectar el efecto del tratamiento en ensayos clínicos. De forma complementaria, Goebl et al. [38] desarrollaron MindGlide, un modelo de *deep learning* entrenado en 4247 exploraciones de RM clínica que extrae de una única secuencia volúmenes regionales y carga lesional. El modelo superó a los algoritmos previos y detectó efectos de tratamiento en ensayos clínicos y en práctica real, abriendo la puerta a reutilizar grandes archivos de RM de práctica clínica hasta ahora infrautilizados.

**Fig. 3.**
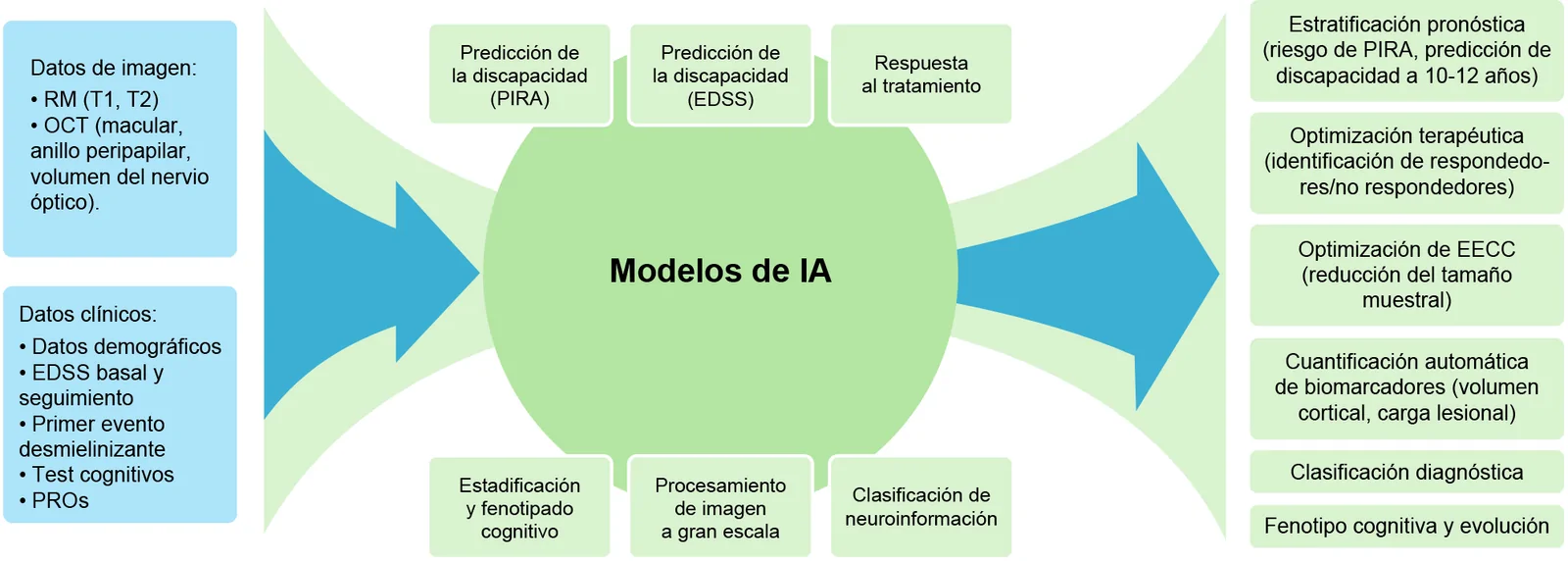
**Uso de modelos de inteligencia artificial en la EM**. Abreviaturas: EECC, ensayos clínicos; IA, inteligencia artificial; OCT, tomografía de coherencia óptica (*optical coherence tomography*); PROs, resultados comunicados por los pacientes (*patient-reported outcomes*).

Un modelo de *deep learning* basado en transformadores generativos, Delphi‑2M, pudo predecir las tasas de más de 1.000 enfermedades y simular trayectorias de salud a 20 años vista a partir del historial médico, edad, sexo, estilo de vida y sin necesidad de biomarcadores [39]. Todos estos resultados sugieren que la IA podría contribuir, en el futuro, a una medicina más predictiva y personalizada. Sin embargo, la implementación de la IA en la práctica clínica debe de hacerse teniendo en cuenta la perspectiva del paciente ya que los avances en IA deben desarrollarse “con” los pacientes, no simplemente “para” ellos [40]. La Fig. 3 resume cómo distintos datos clínicos y de imagen se integran en modelos de IA para mejorar la clasificación diagnóstica, predecir discapacidad y respuesta al tratamiento o identificar fenotipado cognitivo [36,37,38,41,42].

## 9. Mecanismos de Remielinización

**Fig. 4.**
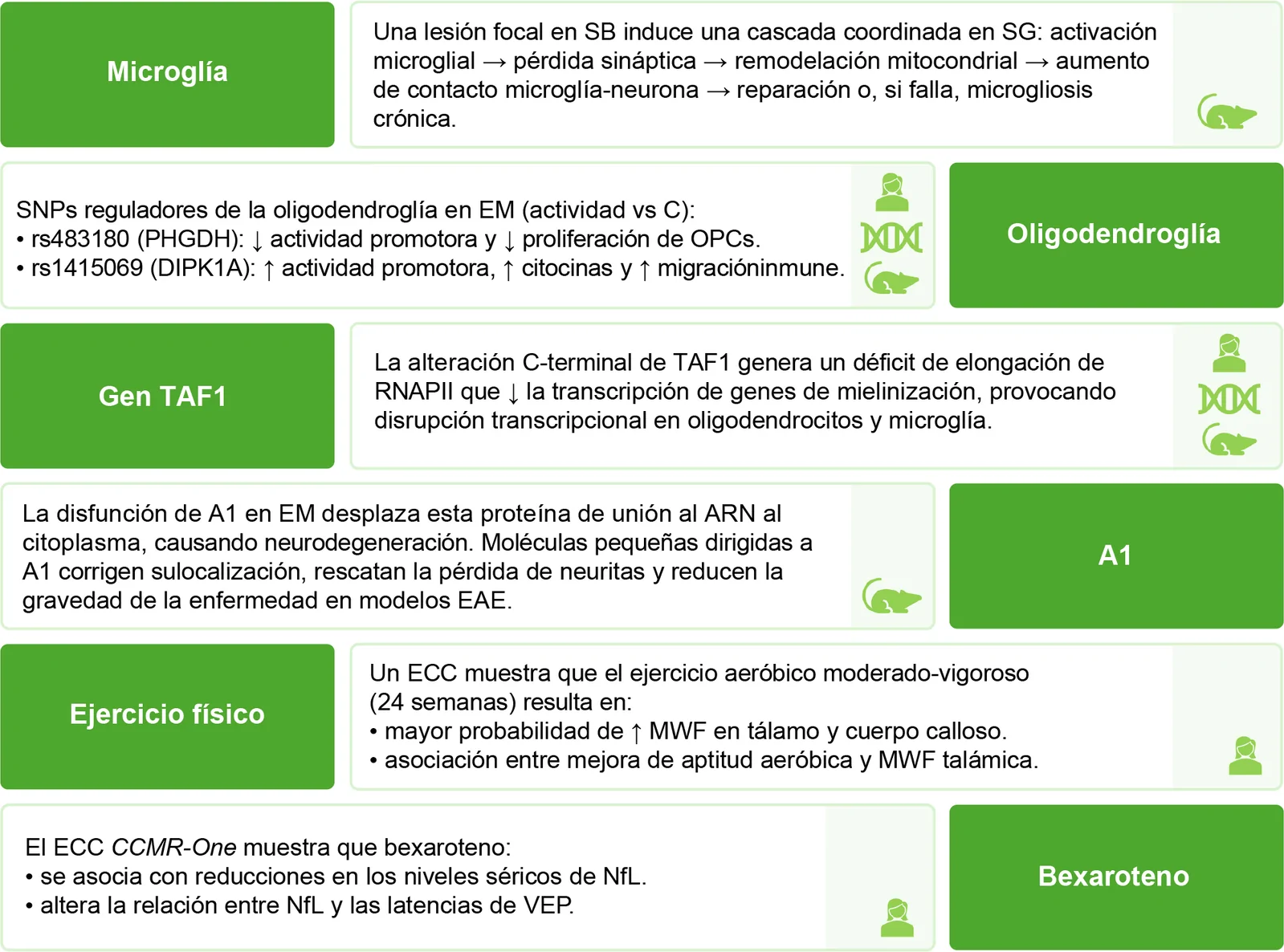
**Mecanismos de remielinización**. Abreviaturas: ARN, ácido ribonucleico; EAE, encefalomielitis autoinmunitaria experimental; ECC, ensayo clínico controlado; MWF, fracción de agua de mielina (*myelin water fraction*); NfL, neurofilamentos de cadena ligera (*neurofilament light chain*); OPCs, células precursoras de oligodendrocitos (*oligodendrocyte precursor cells*); SNPs, polimorfismo de un solo nucleótido (*single nucleotide polymorphism*); VEP, potencial evocado visual (*visual evoked potential*); TAF1, *TATA-box binding protein associated factor 1*; CCMR-One, *Cambridge Centre for Myelin Repair One*. →, conduce a; ↓, disminución; ↑, aumento.

La Fig. 4 resume los resultados sobre los mecanismos de remielinización derivados de estudios tanto en la EAE como en pacientes con EM [43,44,45,46,47,48], destacando el potencial del ejercicio físico y de bexaroteno como potenciales tratamientos reparadores.

Respecto al ejercicio físico, se presentaron los datos procedentes de un ensayo clínico piloto aleatorizado que exploró el impacto del ejercicio aeróbico de intensidad moderada a vigorosa sobre procesos de remielinización en personas con EM (n = 16) y controles (n = 16). Tras un programa de 24 semanas (70–80% de la frecuencia cardíaca máxima), el ejercicio se asoció con una mayor probabilidad de incremento de la fracción de agua de mielina (MWF, *myelin water fraction*), particularmente en el tálamo y el cuerpo calloso. Además, el aumento de la MWF talámica mostró una asociación con la mejora de la aptitud aeróbica, medida mediante el VO_2_máx, lo que sugiere una relación dosis–respuesta entre el acondicionamiento cardiorrespiratorio y los cambios en microestructura mielínica [45]. Por su parte, en el ensayo clínico fase 2a CCMR-One (*Cambridge Centre for Myelin Repair One*), aleatorizado y doble ciego, el grupo tratado con bexaroteno con muestras disponibles (n = 15) mostró una correlación significativa entre los niveles de NfL y las variaciones en la latencia del potencial evocado visual P100, una asociación no observada en el grupo placebo (n = 12). Estos hallazgos sugieren que la remielinización inducida por bexaroteno podría asociarse a una reducción del daño axonal. No obstante, el carácter exploratorio del análisis y el tamaño muestral limitado subrayan la necesidad de incorporar NfL en futuros ensayos de remielinización para validar estas observaciones [48].

En la Fig. 4 se sintetizan, además, los hallazgos sobre el efecto de diferentes mecanismos en la remielinización, como la activación microglial y la perturbación sináptica inducidas por la lesión, la influencia de variantes genéticas sobre la maduración oligodendroglial, la alteración de programas transcripcionales por disfunción de TAF1 (*TATA-box binding protein associated factor 1*) y las moléculas pequeñas dirigidas a A1 [43,44,46,47].

## 10. Biomarcadores

### 10.1 Perspectivas Actuales

Los biomarcadores, desde los moleculares y celulares hasta los de imagen y digitales, tienen aplicaciones potenciales en el diagnóstico, el pronóstico y la monitorización de la enfermedad. Sin embargo, su traslación a la práctica clínica sigue siendo compleja, condicionada por la heterogeneidad biológica, la variabilidad entre plataformas analíticas, la necesidad de estandarización y validación clínica, y las barreras regulatorias y de reembolso. Estas limitaciones explican la brecha persistente entre el descubrimiento de biomarcadores en el ámbito de la investigación y su uso rutinario en la clínica [49].

**Fig. 5.**
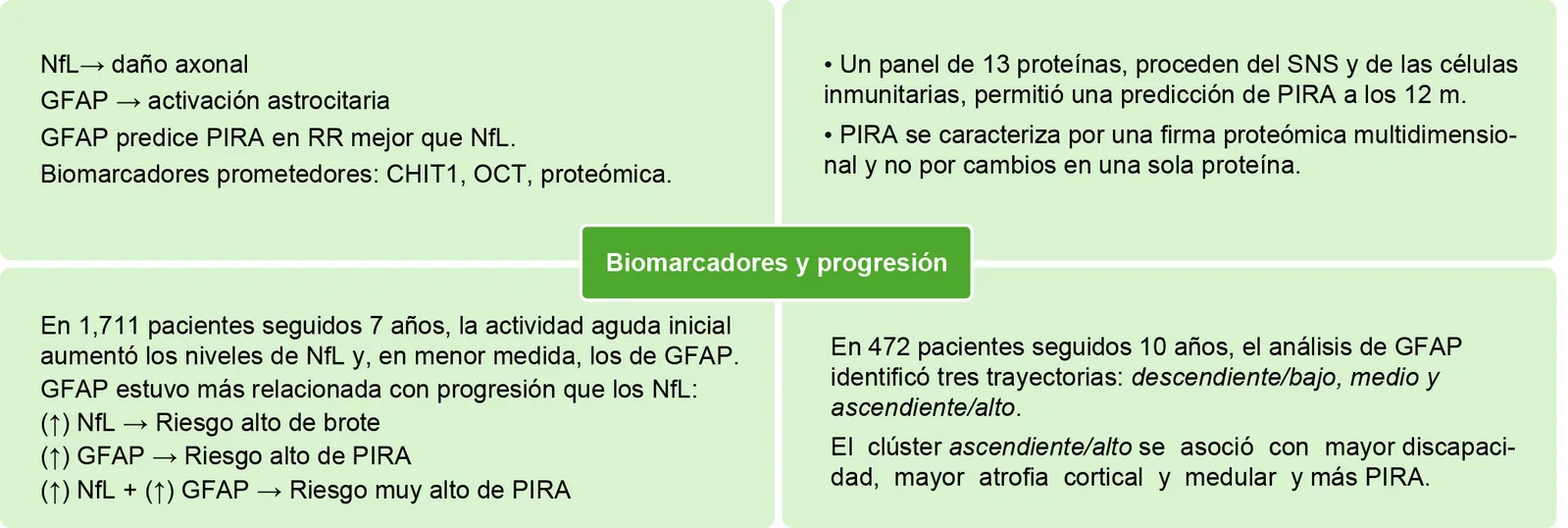
**Biomarcadores y progresión**. Abreviaturas: CHI1, quitinasa 1 (*chitinase 1*); GFAP, proteína ácida fibrilar glial (*glial fibrillary acidic protein*); m, meses; RR, recurrente remitente. →, conduce a; ↑, aumento.

La Fig. 5 muestra las conclusiones de varios estudios recientes sobre biomarcadores de fluidos corporales. De forma resumida, los datos confirman que los NfL se asocian principalmente con daño axonal y el empeoramiento asociado a los brotes (RAW, *relapse-associated worsening*), mientras que la GFAP refleja activación astrocitaria y se asocia con PIRA, especialmente en EM recurrente-remitente (EMRR) [50,51]. La aproximación multimodal, que integra proteómica, OCT y análisis longitudinal de GFAP, pone de manifiesto que la progresión está caracterizada por firmas biológicas múltiples [50,52]. Los pacientes con GFAP ascendiente/alta a lo largo de los años muestran fenotipos con mayor riesgo de discapacidad y atrofia a largo plazo, apoyando su valor como biomarcador de estratificación pronóstica [53].

### 10.2 Monitorización del Tratamiento

Los NfL se presentan como un potencial biomarcador para evaluar la eficacia de los TME, ya que correlaciona con los brotes y la discapacidad [54]. De hecho, se plantea incluir los NfL como endpoint principal en los ensayos fase 2 y como secundario en los fase 3. Los NfL podrían ayudar a evaluar la dosificación de ciertos TME y la posible retirada de estos tratamientos. Se ha planteado que podría ser el momento de incluirlos en la práctica clínica, aunque habría que estandarizar el tipo de medida a usar (*z score* o puntuación total) [55]. En este sentido, un estudio ha mostrado que los z-scores de NfL superaron de forma consistente a la puntuación total (*cut-offs*), dado que captaron mejor la variabilidad interindividual, identificaron actividad subclínica y mostraron mayor capacidad predictiva de lesiones T2 y brotes (Tabla 4, Ref. [56,57,58,59]) [56].

**Tabla 4.** **Efecto de los tratamientos en los biomarcadores**.

Referencia	Objetivo	N	Resultados
Hagler et al. [57]	Evaluar el impacto de los anti CD20 en NfL y GFAP	106 EMRR	A los 12 meses, los pacientes tratados con anti-CD20 muestran un descenso de NfL, pero no se observa cambio significativo en GFAP.
Trogu [58]	Medir el efecto de los espaciados de dosis de rituximab en NfL y GFAP	Cohorte transversal (1217; 97% EMRR)	Los niveles NfL se mantienen estables los primeros 24 meses de la última dosis de rituximab y a partir de ahí ascienden de forma modesta pero significativa.
		Cohorte longitudinal (319 EM)	
			GFAP no mostró variaciones en función del tiempo desde la última dosis de rituximab. Sin embargo, se asoció con EDSS más alta y con una mayor exposición acumulada a rituximab.
			Los NfL se mantienen estables con intervalos de dosificación de rituximab ≤18 meses, independientemente del recuento de células B.
			GFAP no mostró cambios en función del intervalo de dosificación.
Fernández-Vicente et al. [59]	Identificar si hay una asociación entre GFAP tras tratamiento y PIRA	352 EMRR basal, 342 tras 1 año	La GFAP no mostró cambios globales significativos tras un año de tratamiento. Sin embargo, la ausencia de reducción de GFAP se asoció con un mayor riesgo y un tiempo más corto hasta PIRA, incluso tras ajustar por actividad en RM, duración de la enfermedad, tipo de TME y cambios en NfL.
			Los incrementos de GFAP eran mayores cuando NfL permanecía elevado.
			La combinación de GFAP con scores de respuesta al tratamiento (Rio, mRio, MAGNIMS) mejoró la capacidad predictiva de PIRA frente a los scores por separado.
Einsiedler et al. [56]	Comparar valores de NfL estandarizados (z-scores/percentiles ajustados por edad e IMC) vs valores absolutos para la monitorización de la respuesta al tratamiento y la predicción de actividad clínica y radiológica	447 EMR	Los z-scores de NfL superaron de forma consistente a los *cut-offs* absolutos; captaron mejor la variabilidad interindividual, identificaron actividad subclínica y mostraron mayor capacidad predictiva de lesiones T2 y brotes, especialmente en pacientes más jóvenes.
			Además, proporcionaron puntos de corte más precisos para monitorizar respuesta terapéutica.

Abreviaturas: CD, grupo de diferenciación (*cluster of differentiation*); EMRR, esclerosis múltiple recurrente-remitente; IMC, índice de masa corpora.

Los NfL combinados con la GFAP, podrían ayudarnos a predecir la evolución de la discapacidad. Los pacientes con más riesgo de progresión serían aquellos con los niveles de GFAP y NfL altos. Un estudio con 368 pacientes mostró que la reducción de GFAP con TME de alta eficacia y moderada eficacia era muy similar y mínima (3%), al contrario de lo que ocurre con los NfL (48% alta-eficacia vs 34% moderada-eficacia, aunque esta diferencia no fue estadísticamente significativa) [60].

Otros biomarcadores como la proteína similar a la quitinasa 3 tipo 1 (CHI3L1, *chitinase-3-like protein 1*) también se relacionan con la respuesta a algunos tratamientos. Por ej, CHI3L1 predijo actividad de la enfermedad en pacientes tratados con cladribina [61]. Es importante destacar, que, dado que existen multitud de mecanismos de daño en la EM, los paneles que incluyan análisis multivariantes de los biomarcadores serán más precisos que el uso de un solo biomarcador [62].

La Tabla 4 resume los trabajos presentados sobre cambios en los niveles NfL y GFAP tras el tratamiento con anti-CD20 y tras dosis extendida de rituximab [57,58], así como el valor predictivo de GFAP y de los cambios inducidos por los tratamientos en el riesgo de PIRA [59].

## 11. Conclusiones

En el ámbito de la patogenia, la heterogeneidad endotelial regional, la regulación molecular del tráfico leucocitario y la polarización diferencial de los fagocitos del SNC configuran un marco fisiopatológico integrado que sustenta las terapias actuales y orienta la búsqueda de nuevas dianas en la EM. En relación con el VEB, la evidencia respalda su papel como factor de riesgo necesario para el desarrollo de la enfermedad, aunque persisten incertidumbres relevantes: no está aún resuelto si actúa como desencadenante (*trigger*) o como conductor permanente (*driver*) de la EM, no se dispone de ninguna terapia dirigida al virus con eficacia probada en humanos, y queda por dilucidar si la prevención requeriría evitar la propia infección por VEB o bastaría con prevenir la mononucleosis infecciosa.

En el diagnóstico de la NMOSD, los criterios IPND 2025 priorizan el LCBA como técnica de referencia, incorporan el cerebelo como región típica e introducen una clasificación dual que distingue enfermedad seropositiva (AQP4-IgG+) de los síndromes seronegativos, lo que aporta un marco más refinado para el manejo de las formas doble seronegativas.

En el ámbito de la neuroimagen, los datos revisados destacan tres aspectos prácticos para el clínico: la aplicación sistemática de los protocolos estandarizados MAGNIMS-CMSC-NAIMS de RM cerebral, medular y de nervio óptico en el marco de los criterios de McDonald 2024; el valor diagnóstico, pronóstico y de monitorización de las PRLs, las SELs y el CVS cuando se dispone de secuencias sensibles a susceptibilidad magnética optimizadas y de experiencia interpretativa; y la utilidad de la imagen medular cuantitativa para refinar el pronóstico y, en determinados casos, complementar la monitorización con RM cerebral. La IA aplicada a la RM muestra un enorme potencial para predecir la progresión, estimar el efecto individual del tratamiento y optimizar el diseño de ensayos clínicos, si bien su traslación a la práctica clínica requerirá validación externa, integración con la perspectiva del paciente y un desarrollo “con” los pacientes, no solo “para” ellos.

Los avances en mecanismos de remielinización, incluyendo el ejercicio aeróbico y bexaroteno como estrategias reparadoras en estudios exploratorios, refuerzan el valor de incorporar biomarcadores como los NfL en futuros ensayos para validar el daño axonal y la neuroprotección.

Por último, los biomarcadores de fluidos corporales confirman la utilidad complementaria de los NfL (daño axonal y RAW) y de la GFAP (activación astrocitaria y PIRA), y apuntan a que las aproximaciones multivariantes y longitudinales superarán a los biomarcadores aislados en la estratificación pronóstica y en la monitorización del tratamiento.

En conjunto, estos avances reflejan una evolución hacia una EM cada vez más predictiva, multimodal y personalizada, con implicaciones para el diagnóstico precoz, la individualización terapéutica y el diseño de ensayos clínicos.

## Contribuciones de los Autores

ÓF, XM, AArés, EA, YA, AAlon, RA, LB, CC, ABC, TCT, LCF, SE, LL, ML, SL, JEML, VML, EMonr, EMora, COG, JMP, LRP, ÀR, ARA: conceptualización, investigación, redacción—revisión & edición. Todos los autores leyeron y aprobaron la versión final del manuscrito. Todos los autores participaron suficientemente en el trabajo y aceptaron responsabilizarse de todos sus aspectos.

## Aprobación Ética y Consentimiento Informado

No aplicable.
